# A Preliminary In Vitro Study of 3D Full-Field Strain Distribution in Human Whole Premolars Using Digital Image Correlation

**DOI:** 10.3390/ma15062246

**Published:** 2022-03-18

**Authors:** Qing Liu, Qianqian Dong, Yifeng Wen, Baoquan Shi

**Affiliations:** 1 Key Laboratory of Shaanxi Province for Craniofacial Precision Medicine Research, College of Stomatology, Xi’an Jiaotong University, Xi’an 710004, China; qiamqian@163.com (Q.D.); wyf320@connect.hku.hk (Y.W.); 2 Clinical Research Center of Shaanxi Province for Dental and Maxillofacial Diseases, College of Stomatology, Xi’an Jiaotong University, Xi’an 710004, China; 3 Department of Cariology and Endodontics, College of Stomatology, Xi’an Jiaotong University, Xi’an 710004, China; 4 School of Mechano-Electronic Engineering, Xidian University, Xi’an 710071, China; bqshi@xidian.edu.cn

**Keywords:** human premolars, full-field measurement, buccal surface strain, digital image correlation

## Abstract

Full-field measurements can provide a more complete description of the behavior of human whole tooth under load. To that end, in vitro experiments were carried out to measure the full-field buccal surface strains of human premolars free of caries and abrasion using digital image correlation (DIC). Experimental results show that both the value field and the orientation field of strains can be observed exactly, both of which contain a wealth of information. Furthermore, the strain distributions between the crown and the root of specimens were significantly different (*p* < 0.001). An interesting observation was a watershed at the cementoenamel junction (CEJ) which separates the orientation field of strains into two distinct parts; the watershed was also observed in the value field of strains in some specimens whose geometries changed obviously at the CEJ. Another interesting observation was that the minor strains increased linearly from cervical to apical regions in the root cementum. Experimental results also support the viewpoint that mechanisms of non-carious cervical lesions (NCCLs) may in part be due to the changing orientation of tensile strains, as well as their magnitude, and they also support the hypothesis that occlusal force can contribute to root fractures.

## 1. Introduction

Human teeth function under mastication load [1]. The manner in which the resulting strain is distributed is related to their structures [2]. However, their structures are often complex, being hierarchical in that they change at different length scales [3]. They are also naturally occurring graded materials, as their composition, structure, and mechanical properties may vary continuously or in discrete steps, from one location to another [4]. Therefore, understanding the relations between the strain distribution of the whole tooth and its structure is a challenge [5]. Pointwise measurements, such as those provided by strain gauges [6], are sometimes not sufficient to fully monitor an experiment. Bioengineering tools [7] such as finite elements analysis (FEA) [8,9,10,11,12,13], moiré interferometry (MI) [14,15,16,17], electronic speckle pattern interferometry (ESPI) [18,19,20,21], and DIC [22,23,24,25,26,27,28,29,30] have been widely used in studying the mechanics of tooth or dental material.

FEA is a powerful tool for biomechanical analyses in biological research [8,9,10,11,12,13], especially in studying mechanics of tooth or dental material [9]. Matuda et al. [12] used FEA to evaluate the stress distribution in five different class II cavities of premolar models restored with conventional or bulk-fill flowable composite. Palamara et al. [13] used FEA to investigate the effects of load on the location and magnitude of cervical strains of a lower second premolar and a lower central incisor. They found that strains were concentrated at the mid-buccal CEJ for the premolar model regardless of loading direction, and, from oblique loads, vector plots of both models showed tensile vectors in vertical directions, while vertical loads showed tensile vectors in horizontal directions. Lastly, they drew the conclusion that the mechanisms of NCCLs may in part be due to the changing orientation of tensile strains, as well as their magnitude.

MI requires the application of a grid of parallel lines to the surface of the sample to produces moiré fringes, from which the two components of displacement at each point on the surface can be determined [14]. Differentiation of the displacement fields makes it possible to also determine the surface strain tensor. Wang and Weiner [15] employed MI to measure the surface strains on slices of five human premolars. Their study showed that much of the strain is taken up in a soft zone that separates the stiff outer enamel layer from the crown dentin. Kishen et al. [16] used MI to evaluate the biomechanical basis of NCCLs by examining the strain in the enamel and dentine. They observed that the strains in the enamel increased at the cervical edge above the CEJ, while the strains in the dentine increased below the CEJ. The limitation of MI is that it can only measure in-plane displacements or strains [17].

ESPI is a type of laser scanning method that projects electronic speckle patterns on specimens [18]. The speckle pattern changes when the specimen is loaded. The differences between patterns of images obtained before and after applying load are used to detect shifts in the phases of electronic speckle patterns. These phase differences correspond to surface displacements [19]. Zaslansky et al. [20] used ESPI to measure the relative magnitudes of 3D displacements of the outer surface of human premolars. In their study, high values of strain were found mainly near the edge of the enamel cap and were pronounced on the buccal lower region of the tooth. Fages et al. [21] studied the mechanical behavior of the dentin–enamel junction (DEJ) and the dentin–ceramic junction (DCJ) of lower first premolars using ESPI. The limitations of ESPI are the sensitivity to rigid body motion and decorrelation noise affecting the spatial resolution.

DIC is a simple, accurate, noncontact, and full-field deformation measurement method [22], which is widely used in biomedical engineering and material sciences [23,24]. DIC techniques can be further divided into three main categories [25]: two-dimensional (2D) DIC, three-dimensional (3D) DIC, and digital volume correlation (DVC). Two-dimensional DIC uses a single camera measuring in-plane full-field displacements and strains. Three-dimensional DIC is able to measure 3D full-field displacements and strains. DVC can retrieve internal displacements and strains. DIC has proven to be a potential tool that can replace the existing complex sensor devices in dental tests [26]. Wang et al. [27] used 2D DIC to measure the strain distribution of a beam specimen extracted from a sound human third molar. Goellner et al. [28,29] adopted the commercial 3D DIC measurement system Aramis to measure 3D full-field tooth displacement under load in vivo. Zhou et al. [30] adopted DVC to observe the 3D full-field strain in bone–tooth constructs. Although DIC can be used to measure full-field displacements and strains, yet very few applications have been made to natural human teeth, especially to human whole premolars.

The purpose of this study was to quantify the 3D full-field buccal surface strains of natural human premolars under occlusal forces using 3D DIC, particularly to examine the orientation field of strains, which is difficult to observe using other measurement approaches. Furthermore, Palamara et al. [13] found that both the magnitude and the orientation of strains would change at the CEJ, which might contribute to NCCLs. The phenomenon of changes in the magnitude of strains at the CEJ has been observed in previous experiments [16,20]; hence, it was the aim of this study to observe whether the orientations of strains at the CEJ change under occlusal forces in in vitro conditions to further verify the hypothesis put forward by Palamara et al. [30] on the basis of FEA. The null hypothesis was that the orientations of strains at the CEJ would remain constant under occlusal forces.

## 2. Materials and Methods

### 2.1. Specimen Preparation

**Figure 1:**
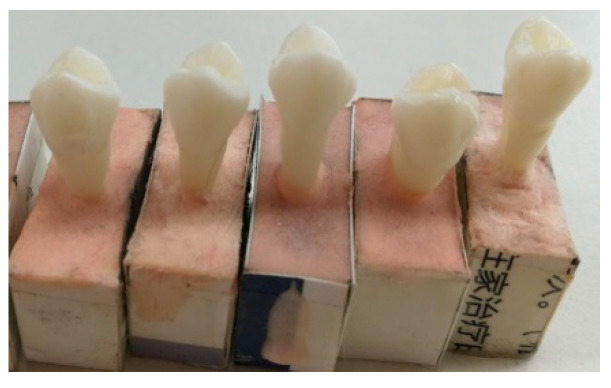
Some prepared specimens in cardboard boxes.

Ten freshly extracted human premolars (half of the maxillary premolars and half of the mandibular premolars from patients aged 18–35 years), free of caries and abrasion, were collected and stored immediately in Hank’s balanced salt solution (HBSS) at 4 °C prior to experiments. When preparing the experiment, rectangular boxes (10 mm × 15 mm × 25 mm) were made using cardboards. Then, self-curing resin (NISSIN (Kunshan), Kunshan, China, E = 17,000.28 ± 47.50 MPa, setting time of about 6 min) was poured into the boxes, and the roots of the premolars were embedded until the curing process was completed (Figure 1). Note that only a small piece of the root was embedded into cardboard boxes, whereby most of the root was exposed for measurement purposes. Afterward, the cardboards were removed. The simple substrates for this vitro study were designed according to the suggestion by Dal Piva et al. [31]. They declared that simplified substrates could be used to evaluate the mechanical behavior of dental specimens without periodontal ligaments because of the rigidity of the specimen.

Next, speckle patterns were made onto the buccal surface of specimens. White matte paint was firstly sprayed onto the interested areas of the teeth. After drying, black matte paint was sprayed onto the white matte paint.

### 2.2. Measurement Setup

**Figure 2:**
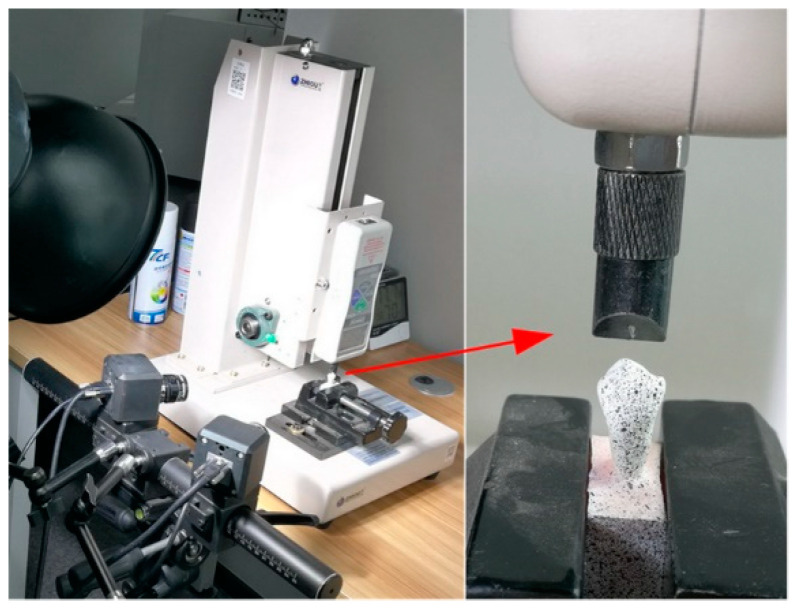
Measurement setup. The 3D DIC device monitoring the buccal surface of the specimen that fixed on the fixture of the testing machine.

The measurement setup consisted of a testing machine (ZHIQU, Dongguan, China) and 3D DIC device (XTOP, Shenzhen, China). As shown in Figure 2, the testing machine was a uniaxial tensile and compression testing machine, whose loading range was 0–100 kg/0–1000 N, with a resolution of ±0.1 kg/±1 N. The 3D DIC device was a commercial displacement and strain measurement system XTDIC, whose accuracy was 0.005%.

During tests, the specimen was fixed on the fixture of the testing machine, and two cameras of the 3D DIC device looked at the buccal surface of the specimen (Figure 2). The load was applied vertically by means of a steel cylinder, and it increased continuously from 0 kg to 60 kg (about two times the normal human tooth chewing force). The cameras captured images (left image and right image) of the specimen synchronously at a rate of 3 fps with a resolution of 3376 × 2704. Note that the two kinds of machines were not synchronized since the testing machine did not provide an interface for the 3D DIC device to gain the load information in real time.

### 2.3. Measurement of 3D Full-Field Buccal Strains

**Figure 3:**
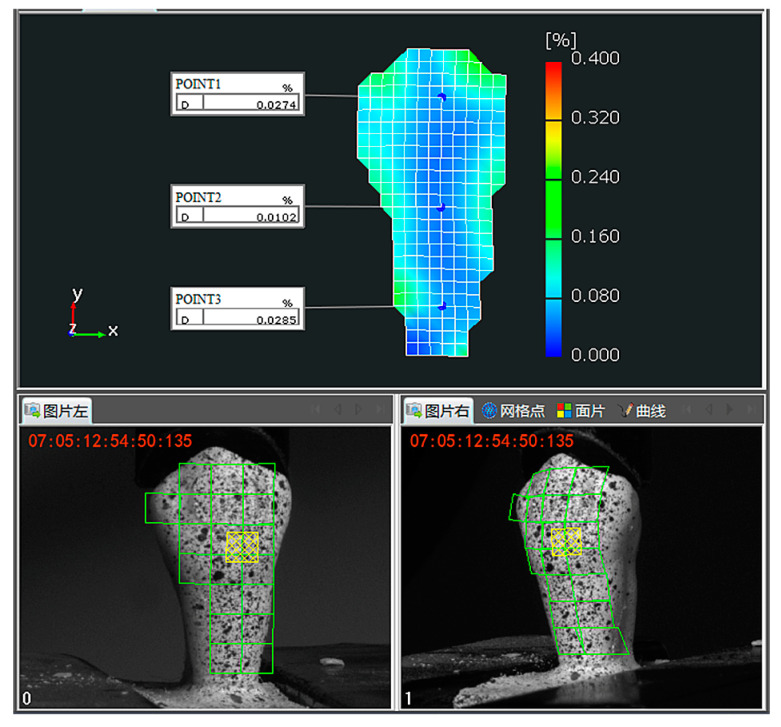
Calculation of full-field buccal surface strains in the software of 3D DIC device. The image marked “0” at the bottom left corner is the image captured by the left camera of the 3D DIC device, while the image marked “1” is the image captured by the right camera of the 3D DIC device. Virtual grids (green square grids) were down-sampled for display purposes.

Once images were captured, as shown in Figure 3, the interested areas were selected manually in the software (XTOP, Shenzhen, China, V8.1) and divided into a series of virtual grids (green square grids down-sampled for better display), whose size was 15 × 15 pixels. Then, the virtual grids within the left image and right image were matched. By connecting the center points of the virtual grids, the surfaces of interested areas were thereby reconstructed. Finally, the 3D full-field strains of the interested areas were calculated according to the dimensional changes between the currently reconstructed surface and the reference surface (the surface reconstructed from the first frames).

### 2.4. Data Evaluation

Strains exported from the 3D DIC device included major strain (or maximum principle strain) and minor strain (or minimum principle strain). The values and directions of strains were derived from the deformation gradient tensors; thus, they were independent of the coordinate system of the 3D DIC device and were universally applicable. Note that a positive strain value indicates stretching deformation (can also be seen as tensile strain), while a negative strain value indicates compressive deformation (or compressive strain). Because the values of major strains measured in this study were positive (except few outliers), while the values of minor strains were negative, the major strain can be seen as tensile strain, while the minor strain can be seen as compressive strain.

Since the load information could not be obtained in real time, the 3D DIC device could not generate the load–strain curve at a point of interest, but provided the stage–strain curve, where a stage refers to a single measurement during the test. According to the stage–strain curve, the strains of any points of interest could be monitored when the force increased gradually.

## 3. Results

### 3.1. Strain Distribution

**Figure 4:**
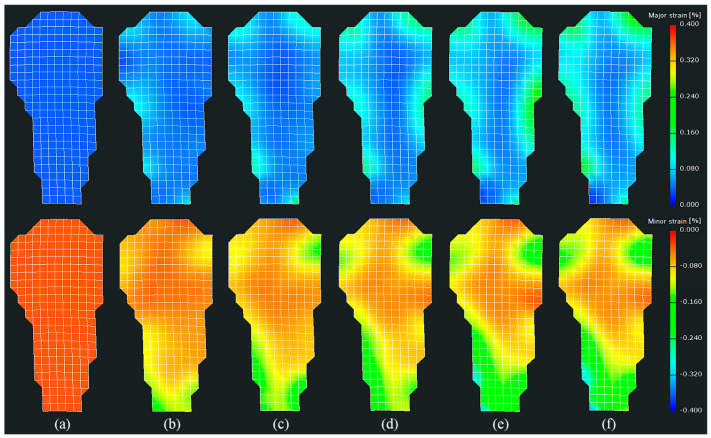
Full-field buccal surface strains of the specimen (shown in Figure 3) at (**a**) stage 0, (**b**) stage 5, (**c**) stage 10, (**d**) stage 20, (**e**) stage 30, and (**f**) stage 39. The first row displays the major strain (or tensile strain) map, while the second row displays the minor strain (or compressive strain) map.

Full-field strain distributions may reveal the whole deformation of the buccal surface of the specimen under load. Typical full-field buccal surface strain distributions of a specimen are shown in Figure 4. For better visualization, only the value field of strains is displayed. It can be seen that the absolute values of either the major strain or the minor strain showed an overall growth trend with the increase in load. In detail, the strains grew substantially before stage 30, before slowing down. Moreover, the growth was inconsistent. The strains in some regions grew rapidly, while those in other regions grew slowly. A clear trend can be seen in the rapid increase in the absolute values of the minor strains in the root of the specimen. It can also be seen that the distributions of either the major strain or the minor strain were complex and inhomogeneous. The distributions of strains in different parts (crown and root) of the specimen were inhomogeneous. Even in the same part, such as the crown, the distributions of strains were also uneven.

**Figure 5:**
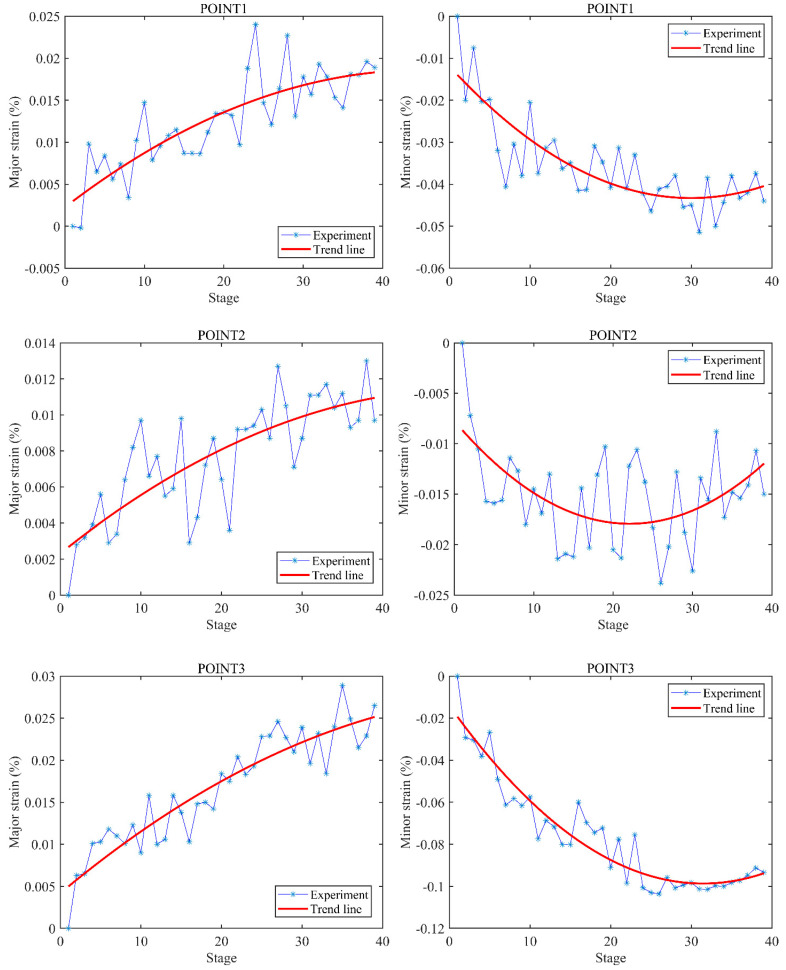
Stage–strain curves of three representative points (POINT1, POINT2, and POINT3, as shown in Figure 3) at the center line on the buccal surface of the specimen. The trend lines were fitted according to the experimental data.

In order to quantitatively observe the changes in the strains with the increase in load, the stage–strain curves of three representative points (POINT1, POINT2, and POINT3) at the center line on the buccal surface within different parts (crown, neck, and root) of the specimen were drawn. As shown in Figure 5, the trend lines show that either the major strain or the minor strain grew approximately linearly in the first 20 stages, before slowing down. An interesting observation is that the growths of the minor strains were almost stagnant upon reaching stage 30. This is consistent with the full-field strain distributions. In addition, the growths fluctuated according to the experimental lines, especially the strains of POINT2 located at the neck of the specimen. It can also be seen that the absolute values of the minor strains were larger than those of the major strains. This indicates that compressive deformations were dominant under occlusion forces.

**Figure 6:**
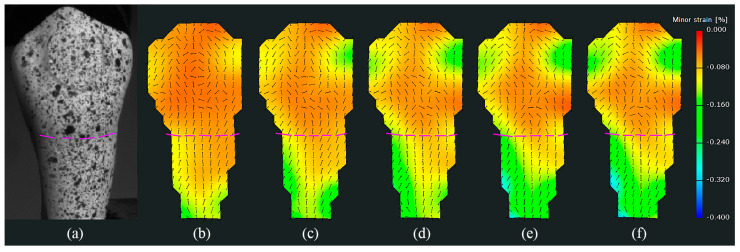
The orientation fields of the minor strains in Figure 4. (**a**) Specimen. (**b**–**f**) Stage 5, stage 10, stage 20, stage 30, and stage 39, respectively. The short thin black lines (unit vector), mapped onto the value fields, represent the directions of minor strains, while the purple dotted lines represent the location of the CEJ.

The orientation field of strains, which is relatively less studied, was also observed. Because the direction of major strain (i.e., the stretching direction) was perpendicular to the direction of minor strain (i.e., the compressive direction) for each point, only the orientation of minor strains was observed. As shown in Figure 6, the unit direction vectors were mapped onto the value field of strains accordingly. It can be seen that the orientation field of strains exhibited different changing patterns compared to the value field of strains with the increase in load. The orientation field exhibited local and irregular variations. Specifically, the directions of strains in the root of specimens were approximately along or perpendicular to the long axis of the tooth and almost unchanged as the load stage increased (the orientation distribution of stage 5 was similar to that of stage 39), whereas the directions of strains in the crown varied from one location to another. A significant observation was a watershed within the neck of the specimen, which separated the orientation field of strains into two distinct parts corresponding to the crown and the root. Upon mapping the orientation field to the physical surface of the specimen, the watershed could be located at/near the CEJ. This finding was also observed in other specimens (as shown in Figures S1–S8 in supplementary materials), illustrating that it is not an accident, but a normal response of the tooth structure under load. In summary, both the orientation field and the value field of strains contain a wealth of information.

**Figure 7:**
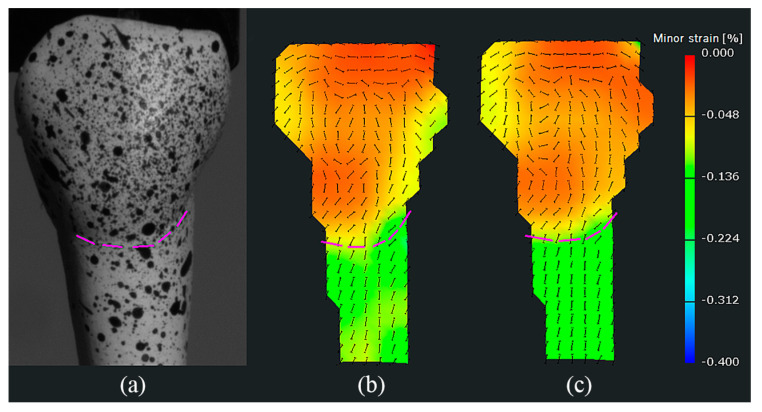
The orientation distributions of the minor strains of (**a**) another specimen at (**b**) stage 15 and (**c**) stage 30. The purple dotted lines denote the CEJ. It can be seen that the watershed exists in both the orientation field and the value field of minor strains.

Additionally, it was found that the watershed not only existed in the orientation field of strains, but also could be seen in the value field of strains in some specimens whose geometries changed obviously at the CEJ. Figure 7 gives an example, where the watershed existed in both the orientation field and the value field of strains. A larger discrepancy of crown–root morphology resulted in a clearer watershed. That is, the distributions of strains were not only dependent on the structures of specimens, but also influenced by their geometries/morphology.

**Figure 8:**
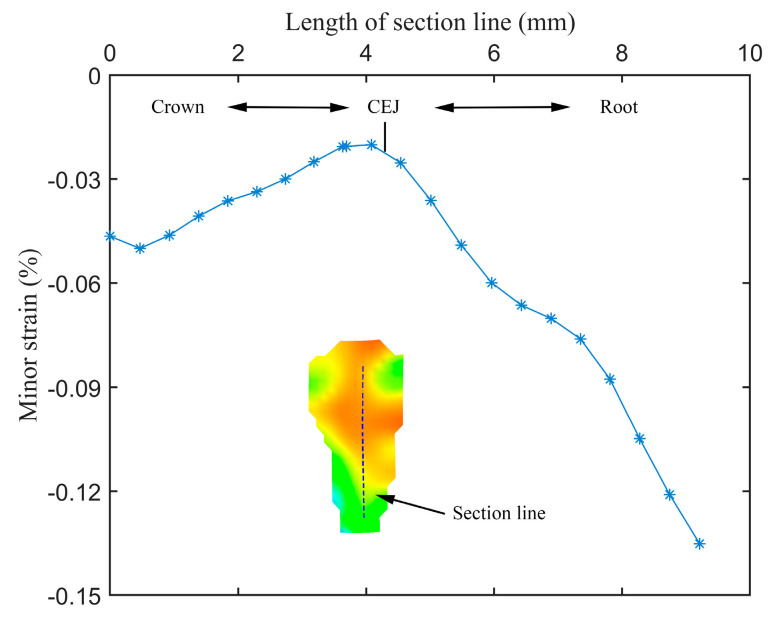
Section line along the center line of the value field of minor strains at stage 39 in Figure 4.

By combining the value field and orientation field of strains, it can be seen that the distribution of strains in the crown was complex and unordered. Not only the values, but also the directions of strains could change with the increase in load. Especially in the region near the CEJ, either the values or the directions of strains could change dramatically. These observations would partially explain why the growths of strains fluctuated in Figure 5. In contrast, the distribution of strains in the root exhibited a certain degree of regularity in either the value field or the orientation field. For a better understanding of the distribution of strains in root, as shown in Figure 8, a section line was created along the center line of the value field of minor strains at stage 39 in Figure 4. It can be observed that the absolute values of minor strains increased almost linearly from the cervical to apical regions in the root surface below the CEJ. In summary, the following conclusions can be drawn: (1) both the values and the directions of strains may change with the increase in load; (2) the value field and the orientation field of strains exhibit different changing patterns as the load increases; (3) the distributions of strains between the crown and the root of specimens are significantly different.

### 3.2. Statistical Analysis

**Figure 9:**
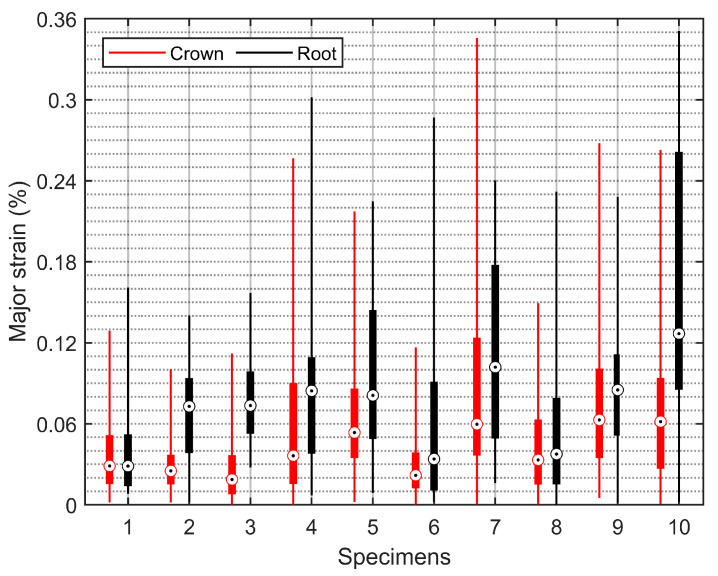
Box plot of the major strains in the crown and the root of specimens. Boxes are composed of the minimum, first quartile, third quartile, median, and maximum strain values.

**Figure 10:**
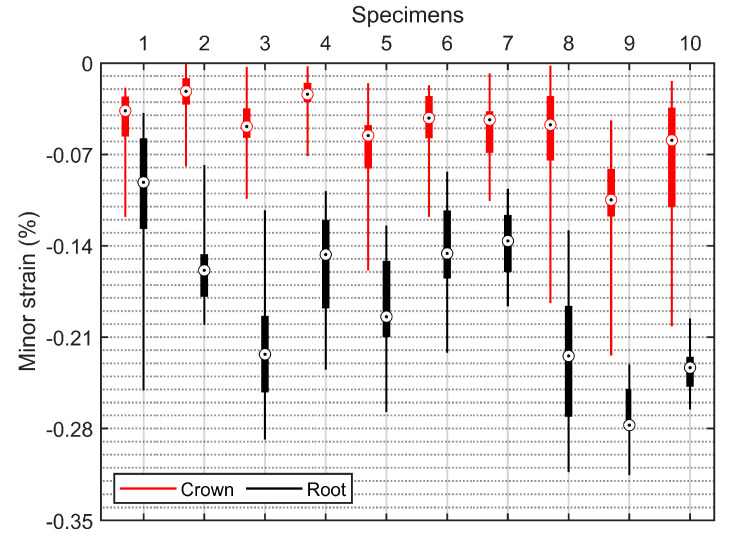
Box plot of the minor strains in the crown and the root of specimens. The composition of the boxes is the same as described in Figure 9.

Statistical analysis was carried out using RStudio (the free desktop version). The buccal surface strains measured at the last stages of the specimens (specimen 1 to specimen 5 were maxillary premolars, while specimen 6 to specimen 10 were mandibular premolars) were analyzed. Note that the strains of edge points and outliers were not counted since they had low confidence. Figure 9 and Figure 10 show box-plot analyses of the major strains and the minor strains in the crown and root, respectively. It can be seen that most of the major strain boxes of the corresponding crown and root overlapped; however, all of the minor strain boxes were distinct from each other, illustrating significant statistical differences in the value field of minor strains between the crown and the root of specimens.

Analysis of variance (ANOVA) was carried out for the mean values of the buccal surface strains. As shown in Table 1, the ANOVA results show that the mean values of the minor strains between the crown and the root of specimens were significantly different (*p* < 0.001). In conclusion, both the visual observation and the statistical analysis show that the distributions of minor strains between the crown and the root were significantly different.

## 4. Discussion

This study showed that 3D DIC can be used to observe the full-field surface strains of human whole tooth. Compared with MI and ESPI, DIC is much less sensitive to ambient vibrations, can detect rigid body motion, and can simultaneously measure full-field displacements and strains [2,5]. Different applications of DIC to dental materials and related fields have been reported in the past [26]. Yet, very few applications have been made to natural human teeth. Goellner et al. [28,29] adopted the commercial 3D DIC measurement system Aramis to measure 3D full-field tooth displacement under load in vivo, but strains were not observed. In this study, both the value field and the orientation field of the buccal surface strains of natural human premolars were accurately measured in vitro using 3D DIC. The value field of strains reflects the magnitudes and the distributions of the (tensile and/or compressive) deformations with the increase in load, while the orientation field reveals the maximum and/or the minimum deformations in specific directions. Moreover, the variation of the strain at any point can be exactly examined according to the stage–strain curve. In brief, 3D DIC can provide rich information, thus helping us to better understand the whole tooth performance under load.

This study also showed that both the orientation field and the value field of strains contain a wealth of information. Previous studies mainly focused on measuring the magnitudes of the strains using a fixed coordinate system [15,20], where the horizontal axis was parallel to the tooth axis, while the vertical axis was perpendicular to the tooth axis; thus, the directions of the strains were only recorded along the axes of the fixed coordinate system. Nevertheless, the directions of strains, outputted from the 3D DIC device, were derived from the deformation gradient tensors; thus, they were independent of the coordinate system of the 3D DIC device and were universally applicable. More specifically, the orientation field of strains showed that the directions of the strains in the root of specimens were approximately along or perpendicular to the tooth axis, whereas the directions of the strains in the crown varied from one location to another. In summary, the orientation field of strains could exactly reflect in which directions the maximum (major) and/or minimum (minor) principal strains occurred.

The value field and the orientation field of strains exhibited different changing patterns as the load increased. The value field of strains showed an overall increasing trend with the increase in load, but the orientation field of strains exhibited local and irregular variations. Specifically, the absolute values of either the major strain or the minor strain increased linearly during the initial stages (could be considered elastic deformation), before slowing down (could be considered plastic deformation) according to the stage–strain maps and curves. This is consistent with the elastic–plastic behavior of a human tooth under compression. Zaytsev and Panfilov [32] presented that human dental enamel is able to consider some elastic (up to 8%) and plastic (up to 5%) deformation under compression, while human dentin exhibits the similar deformation behavior, but the values of its elasticity (up to 40%) and plasticity (up to 18%) are much greater. Although the elastic–plastic behavior of root cementum was not analyzed quantitatively, it can be inferred that the root cementum also possesses elasticity and plasticity since its hardness is much lower than that of crown enamel. With regard to the orientation field of strains, the directions of the strains in root cementum remain almost unchanged, whereas those in crown enamel exhibit local irregular variations, which would result from the complex distributions of enamel rods [33] and the geometry of the crown.

The distributions of strains between the crown and the root of specimens were significantly different. One significant observation was a watershed at the CEJ of the specimen, which separated the orientation field of strains into two distinct parts corresponding to the crown and the root. Moreover, the watershed was also observed in the value field of some specimens whose geometries at the CEJ changed obviously. The differences between both sides of the watershed mainly resulted from the structural differences. The external surface structure of specimens above the watershed is enamel, while that below the watershed is cementum [3]. The Vickers hardness of enamel of premolars is about 394.46 ± 41.92, whereas the hardness of cementum near the neck is about 26.50 ± 7.81 [34]. Hence, even though the enamel may be subjected to higher stress than the cementum, it absorbs most of the applied load due to its greater stiffness, thereby exhibiting smaller deformation [15]. This is also consistent with the statistical results, where ANOVA revealed that the mean values of minor strains were significantly different (*p* < 0.001) between the crown and the root of specimens. Clinically, NCCLs present as a sharp, angular, wedge-shaped lesion in proximity to the CEJ [16]. Palamara et al. [13] explained that mechanisms of NCCLs may in part be due the changing orientation of tensile strains, as well as their magnitude. In this in vitro study, we observed that both the magnitudes and the orientations of the strains could change at the CEJ, which supports the hypothesis put forward by Palamara et al. [13] and again substantiates the role of occlusal load in the etiology of NCCLs.

The 3D full-field strains showed that the strain distribution (either direction or value) in the crown is complex and variable. As mentioned before, the surface structure of the crown is enamel. The mechanical properties of enamel are related to factors including the location, chemical components, and arrangement patterns of the enamel rods [20]. Early studies assumed that enamel was a type of isotropic material. However, researchers have discovered anisotropic mechanical properties through a deeper understanding of tooth structure. The hardness and elastic modulus decrease from the head to tail of the same enamel rod, mostly due to the change in the crystal array directions inside the enamel and organic contents [33]. The material properties vary from one location to another, thus potentially causing inhomogeneity of the strain distributions. Furthermore, the irregular tooth morphology may also contribute to the complex strain distribution in the crown [32].

Another interesting observation is that the minor (or compressive) strains increased linearly from the cervical to apical regions in the root cementum (Figure 8). Chutimanutskul et al. [35] studied the physical properties of human premolar cementum on both buccal and lingual surfaces of the roots at the cervical third, middle third, and apical third. They reported a decreasing gradient in the hardness and elastic modulus of cementum in both surface groups, from the cervical to apical thirds. Yamaguchi et al. [34] measured the hardness and the elastic modulus of cementum at three regions: cervical third, middle third, and apical third. They reported that the mean values of the Vickers hardness of the cementum at the three locations were 26.50, 20.67, and 19.00, respectively, while the mean values of the elastic modulus were 11.67 GPa, 9.13 GPa, and 7.26 GPa respectively. These are consistent with the observations of minor (or compressive) strains increased from the cervical to apical regions. Clinically, root fractures are a relatively frequent dental condition, which may result from occlusal forces, incorrect prosthetic treatment, parafunctional habits, etc. [36]. This study supports the hypothesis that occlusal force can contribute to root fractures since both the major (or tensile) and the minor (or compressive) strains within the root were the largest according to the box-plot analysis (Figure 9 and Figure 10).

Limitations of this study can be analyzed from several aspects. Firstly, only buccal surface strains of the specimens were measured. Previous works have shown that the lingual surface has different mechanical properties from the buccal surface and, thus, different strain distributions [37]. The strain distribution within the lingual surface was not observed since only a 3D DIC device is available. By combining several 3D DIC devices, it is possible to observe the strain distributions of the entire surfaces of specimens. Secondly, the loads in the testing machine were not synchronized with the 3D DIC device in real time since the testing machine did not provide an interface for gaining the load information. Lastly, the roots of specimens were cured in self-curing resin, whereas the real tooth is fixed by periodontal ligament and alveolar bone; thus, there may exist differences in their behavior.

## 5. Conclusions

It may be concluded from this preliminary in vitro study that (1) 3D DIC can be used to observe the full-field surface strains of human whole tooth, (2) both the orientation field and the value field of strains contain a wealth of information, exhibiting different changing patterns with the increase in load, (3) the distributions of the strains between the crown and the root of specimens were significantly different, with an interesting observation of a watershed at the CEJ, which separated the orientation field of strains into two distinct parts, while it was also observed in the value field of strains in some specimens whose geometries changed obviously at the CEJ, and (4) the minor (or compressive) strains increased linearly from the cervical to apical regions in the root cementum. In addition, the experimental results support the following hypotheses in in vitro conditions: mechanisms of NCCLs may in part be due the changing orientation of tensile strains, as well as their magnitude; occlusal force can contribute to root fractures.

## Figures and Tables

**Table 1:** Statistical analysis of strains between the crown and the root of specimens.

Specimen Number	Crown vs. Root	
	Major Strain	Minor Strain
1	*NS*	*
2	*	*
3	*	*
4	*NS*	*
5	*	*
6	*	*
7	*NS*	*
8	*NS*	*
9	*NS*	*
10	*	*

* *p* < 0.001, *NS*: not significant.

## Data Availability

The data presented in this study are available from the corresponding authors upon reasonable request.

## Supplementary Materials

The following supporting information can be downloaded at https://www.mdpi.com/article/10.3390/ma15062246/s1: Figure S1: The orientation distributions of the minor strains of (a) specimen 3 at (b) stage 30, Figure S2: The orientation distributions of the minor strains of (a) specimen 4 at (b) stage 30, Figure S3: The orientation distributions of the minor strains of (a) specimen 5 at (b) stage 30, Figure S4: The orientation distributions of the minor strains of (a) specimen 6 at (b) stage 30, Figure S5: The orientation distributions of the minor strains of (a) specimen 7 at (b) stage 30, Figure S6: The orientation distributions of the minor strains of (a) specimen 8 at (b) stage 30, Figure S7: The orientation distributions of the minor strains of (a) specimen 9 at (b) stage 30, Figure S8: The orientation distributions of the minor strains of (a) specimen 10 at (b) stage 30.
