# Supplementary material for: A Preliminary In Vitro Study of 3D Full-Field Strain Distribution in Human Whole Premolars Using Digital Image Correlation
Source: Materials (Basel). 2022 Mar 18;15(6):2246. doi: 10.3390/ma15062246 (PMC8956105; doi:10.3390/ma15062246)
Supplement: Supplementary file 1 [file materials-15-02246-s001.zip › materials-1616573-supplementary.pdf]

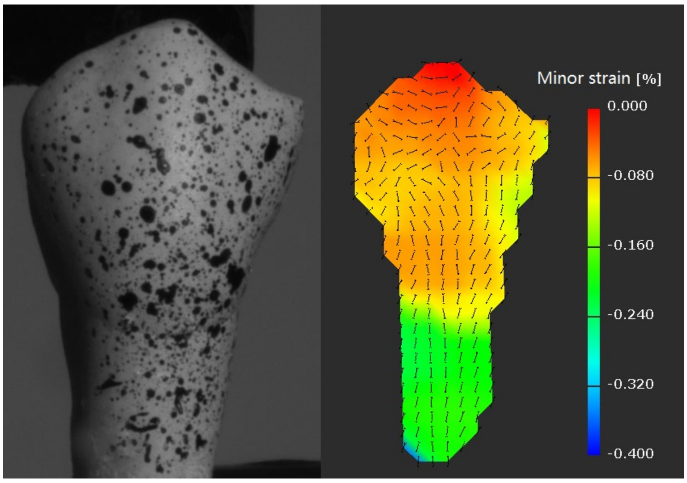

Figure S1 The orientation distributions of the minor strains of (a) specimen 3 at (b) stage 30.

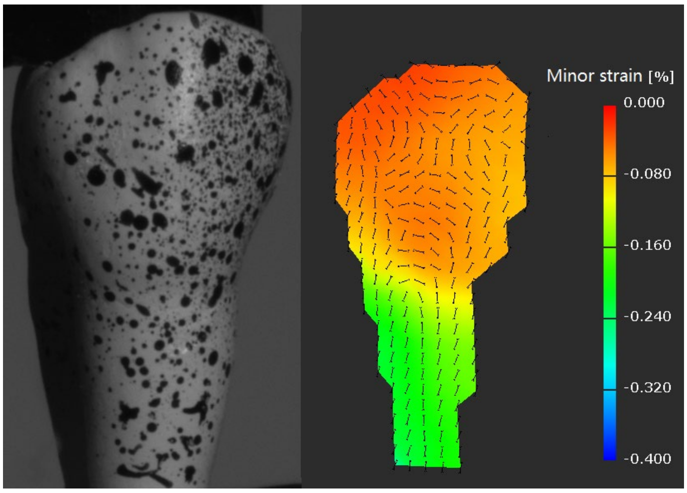

Figure S2 The orientation distributions of the minor strains of (a) specimen 4 at (b) stage 30.

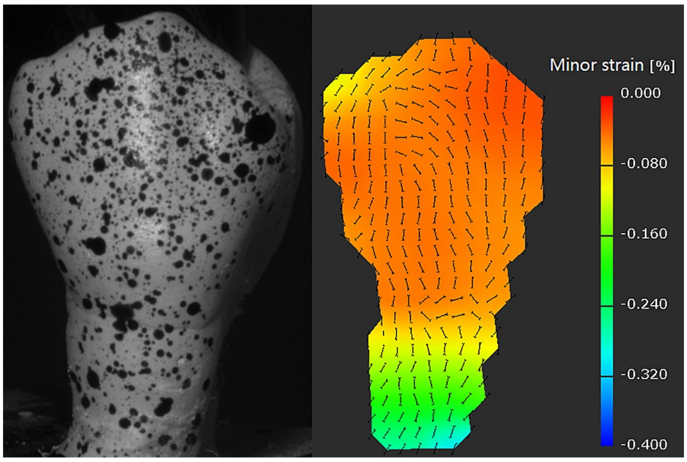

Figure S3 The orientation distributions of the minor strains of (a) specimen 5 at (b) stage 30.

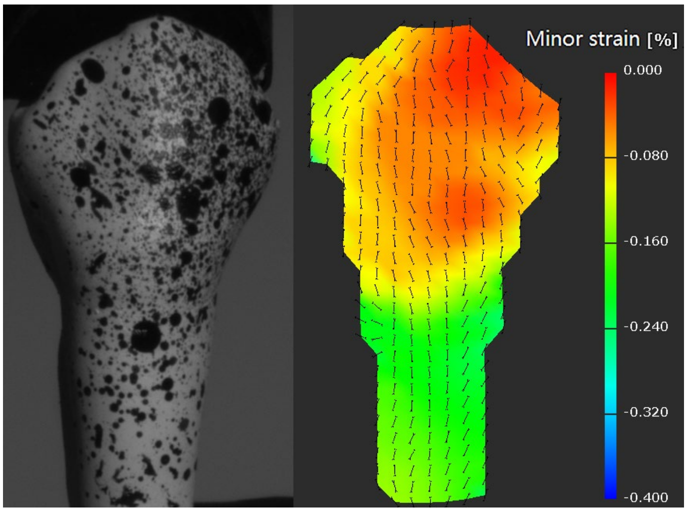

Figure S4 The orientation distributions of the minor strains of (a) specimen 6 at (b) stage 30.

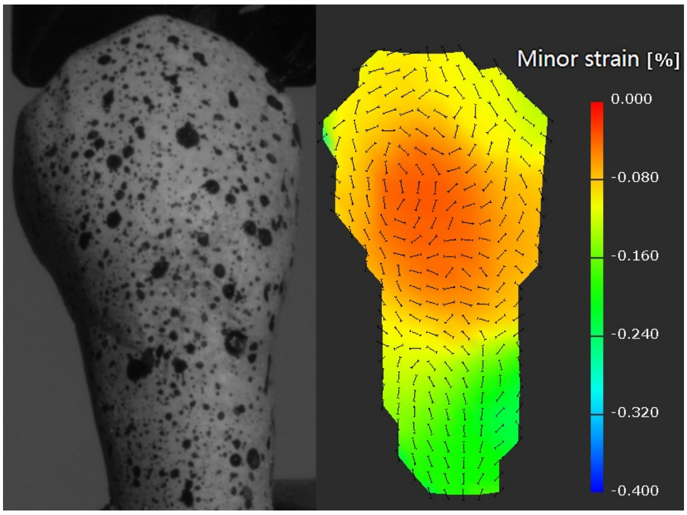

Figure S5 The orientation distributions of the minor strains of (a) specimen 7 at (b) stage 30.

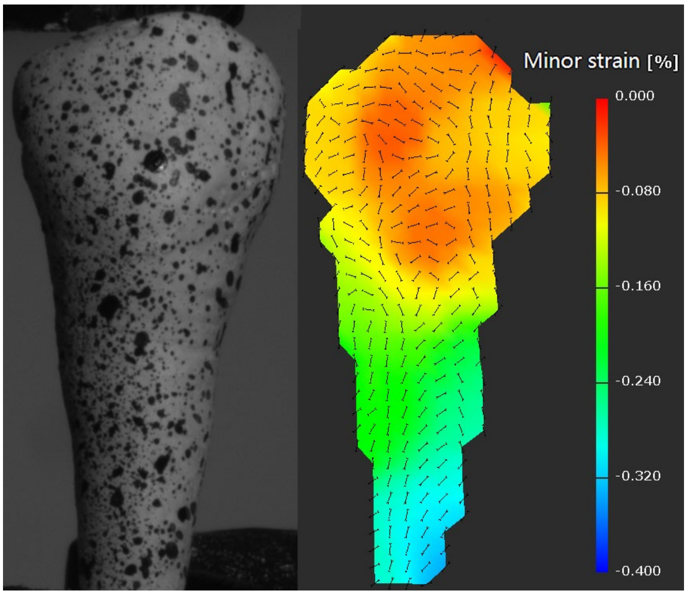

Figure S6 The orientation distributions of the minor strains of (a) specimen 8 at (b) stage 30.

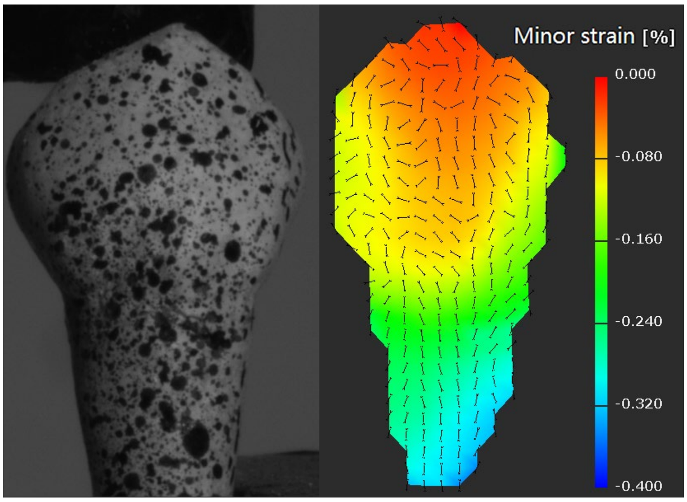

Figure S7 The orientation distributions of the minor strains of (a) specimen 9 at (b) stage 30.

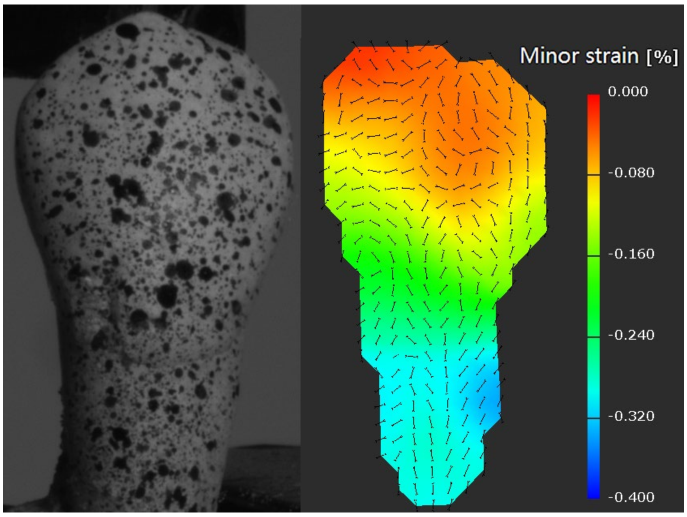

Figure S8 The orientation distributions of the minor strains of (a) specimen 10 at (b) stage 30.
